# My Hand or Yours? Markedly Different Sensitivity to Egocentric and Allocentric Views in the Hand Laterality Task

**DOI:** 10.1371/journal.pone.0023316

**Published:** 2011-08-03

**Authors:** Nuala Brady, Corrina Maguinness, Áine Ní Choisdealbha

**Affiliations:** 1 School of Psychology, University College Dublin, Belfield, Dublin, Ireland; 2 School of Psychology, Trinity College Dublin, College Green, Dublin, Ireland; 3 Institute of Neuroscience, Trinity College Dublin, College Green, Dublin, Ireland; Royal Holloway, University of London, United Kingdom

## Abstract

In the *hand laterality task* participants judge the handedness of visually presented stimuli – images of hands shown in a variety of postures and views - and indicate whether they perceive a right or left hand. The task engages kinaesthetic and sensorimotor processes and is considered a standard example of motor imagery. However, in this study we find that while motor imagery holds across egocentric views of the stimuli (where the hands are likely to be one's own), it does not appear to hold across allocentric views (where the hands are likely to be another person's). First, we find that psychophysical sensitivity, *d*', is clearly demarcated between egocentric and allocentric views, being high for the former and low for the latter. Secondly, using mixed effects methods to analyse the chronometric data, we find high positive correlation between response times across egocentric views, suggesting a common use of motor imagery across these views. Correlations are, however, considerably lower between egocentric and allocentric views, suggesting a switch from motor imagery across these perspectives. We relate these findings to research showing that the extrastriate body area discriminates egocentric (‘self’) and allocentric (‘other’) views of the human body and of body parts, including hands.

## Introduction

The study of mental rotation has a long history in psychology and neuroscience. In 1971 Shepard and Metzler showed that response times to match a pair of three-dimensional shapes – where one is rotated in depth relative to the other - increase linearly with the angle of rotation between the shapes [1]. Response times are symmetric about 180°, being roughly equal for clockwise and counter-clockwise rotations of the same magnitude. This pattern suggests an analog representation: an internal visual image that is mentally rotated in the same way that a real object is physically rotated in space.

**Figure 1:**
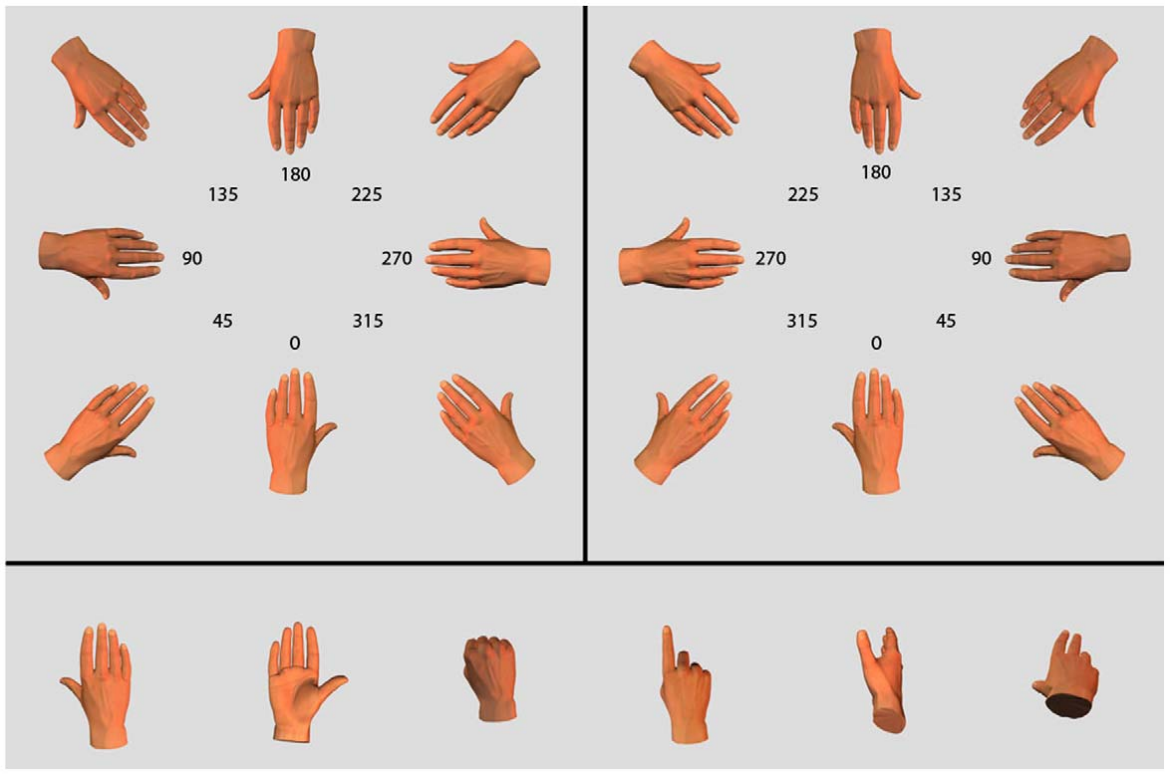
Examples of the stimuli. Images of the back of hand stimulus shown in eight orientations labelled 0° to 315° in clockwise direction for right hand stimuli (upper right) and in anticlockwise direction for left hand stimuli (upper panel). For both right and left hands, ‘medial’ orientations include 45°, 90° and 135°, and ‘lateral’ orientations include 315°, 270° and 225°. The six different hand postures are shown at 0° orientation in the lower panel.

Like objects, bodies and parts of bodies can also be mentally rotated. In the ‘hand laterality task’ participants judge the handedness of visually presented stimuli – images of hands shown in a variety of postures and views as in Figure 1 - and indicate whether they perceive a right or left hand. Response times show a marked non-linearity in this task, increasing more rapidly the further the stimulus is rotated from 0°, and are asymmetric about 180° for many postures. This chronometric signature suggests the engagement of kinesthetic and sensorimotor processes [2]–[4]. The internal image is a motor image of one's own hand that is mentally rotated into the stimulus posture, in the same way that a real hand is physically rotated in space.

In this paper we ask whether the use of motor imagery, which normally assumes a first person perspective, holds for the full range of orientations in the hand laterality task. We find that this is not the case. Motor imagery appears to be used across egocentric views of the hands, but not across allocentric ones.

As shown in Figure 1, hand orientations around 0° are usually depicted in egocentric perspective in the hand laterality task, whereas those around 180° are usually depicted in allocentric perspective. ‘Egocentric’ and ‘allocentric’ are defined as viewpoints that are consistent with the stimulus being an image of one's own hand, or of another person's hand, respectively [5]. Hands in egocentric perspective are visually and motorically familiar, reflecting the use of our own hands. In contrast, images at 135°, 180° and 255° in Figure 1 are motorically unfamiliar in the sense of being awkward or difficult to assume.

Using response time data to distinguish different types of mental imagery or cognitive processes is difficult [6]. We address this issue in two ways. First, we employ a novel measure of performance. Psychophysical sensitivity, *d*', has not, to our knowledge, been previously measured in the hand laterality task. It reveals a clear split in performance for egocentric and allocentric views of the hands. Secondly, we use mixed effects methods to model the response time data [7]. This allows us to look both at *fixed effects* (how average response times vary with hand orientation) and *random effects* (how participants' response times correlate across hand orientation). We show high, positive correlation between response times for pairs of egocentric views, suggesting a common use of motor imagery across these views; but much lower correlation between egocentric and allocentric views, suggesting a shift away from motor imagery across these perspectives.

Behavioural evidence for the use of motor imagery in the hand laterality task comes from the innovative studies of Parsons [4], [8]. His conclusion that the temporal properties and biomechanical constraints of real movement are reflected in motor imagery rests upon a number of findings. Most notably, the time it takes to make a right-left judgment correlates positively with the time it takes to assume a stimulus posture or to imagine rotating one's hand into that posture [4], [8]. The further a stimulus hand moves from a ‘canonical’ position - an orientation requiring minimal hand rotation to match from a resting position with one's hands in the lap - the longer laterality judgments take. Adopting a more awkward resting position, such as by holding one's hands behind one's back, slows response times further, possibly by increasing the length of the ‘mental trajectory’ [8], [9]. Also, for pairs of stimulus postures with the same degree of rotation, those in ‘lateral’ orientation with fingers pointed away from the body's midline (Figure 1) generally take longer to assume, and longer to recognize as right or left hands than those in ‘medial’ orientation [4].

These parallels between mental and biomechanical hand rotation support the idea that we imagine our own hand moving into the stimulus posture to confirm a judgment of handedness, a hypothesis further supported by evidence from neuropsychological [10] and brain imaging studies [11]–[13].

However, the correspondence between mental and physical hand movement varies by stimulus orientation. While response times to make right-left judgments are equivalent to actual movement times for common, easy to adopt postures, response times often exceed movement times for more awkward, hard to adopt postures [8]. As discussed above, an interesting property of many of these ‘awkward’ orientations is that they are seen more frequently from an allocentric than an egocentric perspective; perspectives now known to be distinguished in the visual representation of the human body [5], [14]. Here, using six highly familiar hand postures - views of the back and of the palm of the hand, views of the hand pointing with the index finger, making a fist, reaching to grasp and reaching to shake hands – and eight orientations that include both egocentric and allocentric views of the hand, we measure participants' response times and psychophysical sensitivity, *d*', in judging hand laterality.

## Methods

### Participants

Thirty right-handed participants (13 males), as measured with the Edinburgh Handedness Inventory [15], volunteered to take part in the study. They had a mean age of 26.9 years (SD  = 6.6 years) and a mean handedness index of 97.26 (SD  = 8.84). With the exception of the three authors, all participants were naïve to the purpose of the experiment. All gave written, informed consent consistent with the Declaration of Helsinki, and the study was approved by University College Dublin Ethics Committee.

### Stimuli

Forty-eight images of right hands were created using Poser® 7. These included 6 natural, familiar postures shown in Figure 1, each at 8 orientations ranging from 0° to 315° in steps of 45°. These right hand images were flipped about the vertical axis in Adobe Photoshop® to create mirror symmetric left hand images for a total of 96 stimuli. For the purpose of data analyses, the right-hand stimuli are labelled 0° to 315° running clockwise and the left-hand stimuli are labelled 0° to 315° running counter clockwise. Thus, whether for right or left hands, hands oriented between 45° and 135° point toward the midsaggital plane of the body whereas those between 315° and 225° point away from the midsaggital plane of the body. These correspond to ‘medial’ and ‘lateral’ postures respectively [8]. The images measured 692×602 pixels and subtended ∼25.8×22.4 degrees of visual angle at a viewing distance of ∼60 cm. The stimuli were presented on a Dell Precision 360 PC using Presentation®, which also recorded participants' responses on a Cedrus® RB-530 response box.

### Procedure

Participants sat at a comfortable viewing distance of ∼60 cm from the computer screen where stimuli were presented centrally against a black background. They were asked to identify the laterality of the hand presented on each trial, by pressing the right button of the response box with their right foot or the left button with their left foot in response to a stimulus that they perceived as being a right or left hand respectively. They were asked to respond as quickly as possible as soon as they were reasonably certain of the hand laterality.

After one block of practice trials, with 30 images selected randomly from the full set of 96 images (2 lateralities by 8 orientations by 6 postures), participants completed 9 blocks of 96 experimental trials for a total of 864 trials, with optional breaks between blocks. The 9 experimental blocks were grouped into three consecutive sets of three, with each of three conditions in each set. The participants' own hand postures were varied across conditions. These included a ‘natural’ posture, in which participants rested both hands on their thighs, with their fingers pointing towards the knees; and two ‘unnatural’ postures, in which participants rested their right (or left) hand on their right (or left) thigh while holding their other hand in an awkward posture behind their back. The order of the 3 conditions was counter-balanced across participants, with each participant repeating their assigned order three times. Within every block of 96 trials the hand stimuli were pseudo randomized.

## Results

The data were analyzed in R [16] using linear mixed-effects models [7]. Recursive partitioning [17] was used to further explore the effect of stimulus orientation on both reaction time and sensitivity. The current focus is on how these measures vary with stimulus hand orientation, independently of whether participants are judging right or left hands and independently of the posture of their own hands. Relative to the effect of stimulus hand orientation, the effects of these other manipulations are small [18]; analysis of the response time data using repeated measures ANOVA with factors of *Laterality* (2 levels), *Orientation* (8 levels) and *Condition* (3 levels) shows significant main effects of *Orientation*, F(7,203)  = 62.02, p∼0, *ε* = 0.22, *η^2^_G_* = 0.31, of *Laterality*, F(1,29)  = 15.48, p<0.01, *η^2^_G_* = 0.02, and a significant *Laterality* by *Condition* interaction, F(2,58)  = 7.27, p<0.01, ε = 0.74, *η^2^_G_* = 0.003, where *ε* is the Greenhouse Geisser epsilon and *η^2^_G_* is generalized eta squared [19]. The main effect of *Condition* was not significant, F(2,58)  = 0.04, *p* = 0.96. See reference [18] for further details.

### Reaction Time

Response times less than 450 ms were removed from the raw data as ‘anticipatory errors’, and accounted for 0.44% of the total number of trials. In line with previous studies response times on correct trials that exceeded 3500 ms (4.27%) were also removed [3], [8], [9], [20]. For each participant, and each combination of laterality, orientation, condition and posture, response time (RT) was calculated as the average time of the correct trials out of the 3 repeats.

**Figure 2:**
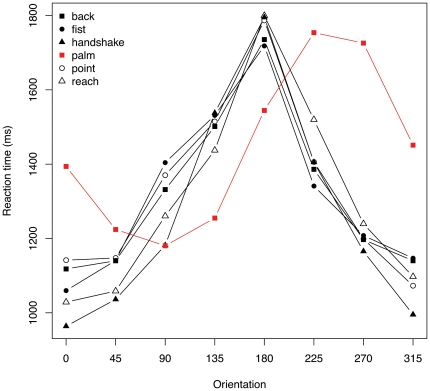
Mean response time by stimulus orientation and posture. Mean response time plotted as a function of stimulus hand orientation for all 6 hand postures. The palm posture, shown in red, is different from the others, being markedly asymmetrical about 180° with higher response times to ‘lateral’ rotations (315°, 270°, 225°) than to ‘medial’ rotations of the same magnitude (45°, 90°, 135°).

A preliminary look at the data reveals that of the six postures used, the palm posture, highlighted in Figure 2, shows an RT-orientation profile that is very different to the others. As previously described for the palm posture [8], [21], the profile is distinctly asymmetric about 180°, RTs being markedly greater for lateral than for medial rotations of the same magnitude. In contrast, for the other 5 postures, the RT-orientation curves are non-monotonic at and roughly symmetric about 180° as previously described for specific, familiar postures such as the back of the hand [8]. Therefore, we exclude the palm posture from the analyses presented below. Including the palm posture in the analyses does not, in fact, change the results. However, as the inclusion of a larger number of postures with RT-orientation profiles similar to the palm posture undoubtedly would change the results, we restrict the analyses to the postures sharing a common RT-orientation profile.

**Figure 3:**
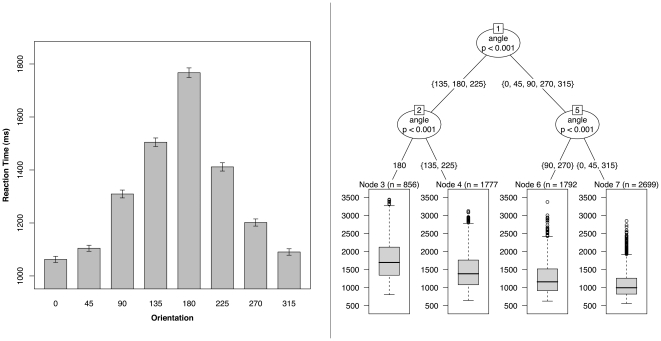
Mean response time by stimulus orientation. (Left panel) Mean response time, calculated across 5 of the 6 postures, plotted as a function of stimulus hand orientation. Error bars show ±1 S.E.M. (Right panel) Classification tree of response time showing a primary split between egocentric (0°, 45°, 90°, 270°, 315°) and allocentric (135°, 180°, 225°) views of the hands.

As shown in the left panel of Figure 3, mean RT, calculated across the 5 remaining postures, increases with angle of rotation from 0°, peaking at 180° and declining again toward 315°. For medial orientations, particularly steep increments in RT occur between 90° and 135° and between 135° and 180°. These are mirrored for lateral orientations so that response times are particularly long for orientations of 135°, 180° and 225°.

The right panel of Figure 3 shows a classification tree of the response time data based on the unbiased recursive partitioning framework of Hothorn and colleagues [17]. This is a two step procedure where the covariate with the highest association with the dependent variable (based on a Strasser-Weber permutation test) is chosen, and this covariate is then split to maximize the difference between the dependent variable in the two subsets. The procedure continues until the p-value of the test for independence between the dependent variable and the covariates, reported at each node, falls below 5 per cent. Intuitively, the first node is the most important one, with successively lower splits having successively less discriminatory power. Here the first split is between egocentric (0°, 45°, 90°, 270°, 315°) and allocentric (135°, 180°, 225°) views of the hand stimuli, Bonferroni corrected *p*<0.001.

**Figure 4:**
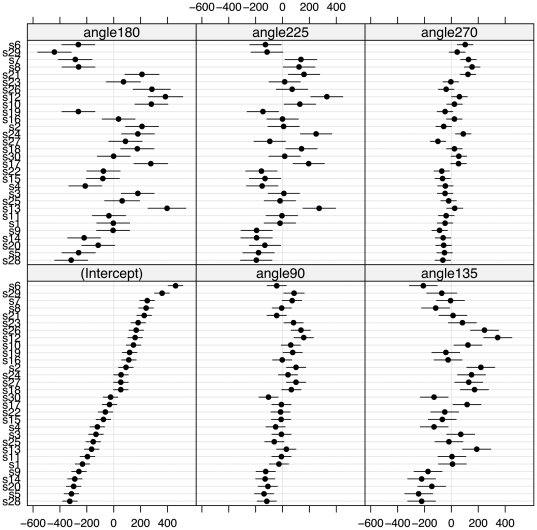
Random effects for response time. Caterpillar plots demonstrating the variability in participants' response times about the fixed effects. In each of the six plots, the thirty participants (labelled s1 to s30 by alphabetical order of their surnames) are ordered, from fastest to slowest, by their response times in the baseline condition (combined 0°, 45°, 315°) or ‘intercept’. The plots show strong correlation between performance in the baseline and the other egocentric orientations (90°, 270°) and weaker correlation between baseline performance and the allocentric orientations (135°, 180°, 225°).

The output of a linear mixed-effects model of response time on hand orientation is shown in Table 1 and in Figure 4. The fixed effects, listed in Table 1, are the averages across participants. They show the estimated increment in response time relative to the ‘intercept’, i.e., the estimated response time for the baseline condition (∼1086 ms) which is set in the model to the combined orientations of 0°, 45° and 315°. Using the criterion of t >2, all other orientations lead to significant increases in response time. The random effects, plotted in Figure 4, show individual variability about the fixed effects.

**Table 1:** Effects of orientation on response time.

	Estimate (S.E.)
(Intercept)	1086.0 (39.4) ***
angle: 90/baseline	226.8 (21.0) ***
angle: 135/baseline	422.7 (32.4) ***
angle: 180/baseline	692.7 (45.3) ***
angle: 225/baseline	328.1 (32.6) ***
angle: 270/baseline	116.2 (19.0) ***
Log-likelihood−51679	
Deviance103408	
AIC103415	
BIC103607	
N7124	
Groups30	

***p<0.001.

In each of the six plots in Figure 4, the thirty participants are ordered from fastest to slowest by their performance in the baseline condition. These plots show that participants' performance is highly correlated with their baseline performance for other egocentric views, the correlation coefficient with the baseline condition being 0.57 for 90° and 0.61 for 270°. Correlation is lower for the allocentric views, with correlation coefficients of 0.28 for 135°, 0.01 for 180° and 0.32 for 225°. In addition, the error bars, which show the standard error of the coefficient estimate for each participant, are considerably longer for 135°, 180° and 225°, reflecting greater trial by trial variability in RT at these orientations.

### Sensitivity, *d*'

**Figure 5:**
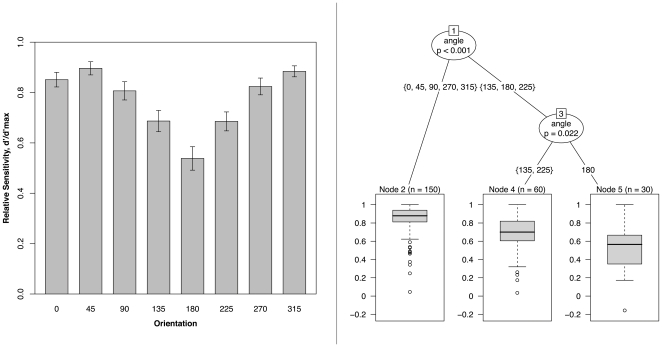
Mean sensitivity by stimulus orientation. (Left panel) Mean relative sensitivity, *d*'*/d*'*_max_*, calculated across 5 of the 6 postures, plotted as a function of stimulus hand orientation. Error bars show ±1 S.E.M. (Right panel) Classification tree of *d*'*/d*'*_max_* showing a primary split between egocentric (0°, 45°, 90°, 270°, 315°) and allocentric (135°, 180°, 225°) views of the hands.

Figure 5 plots *d*' - a measure of sensitivity that is independent of response bias calculated from the proportion of ‘hits’ and ‘false alarms’ [22] - against hand orientation. In this plot *d*' is scaled so that maximum sensitivity is indicated by a value of 1.0. Sensitivity, like RT, changes with angle of rotation from 0°, but here the change is not a gradual one. Rather, *d*'*/d*'*_max_* is high for egocentric views of the hands (0°, 45°, 90°, 270°, 315°) and drops dramatically for allocentric views (135°, 180°, 225°), with sensitivity being particularly low for 180°. The classification tree in the right panel of Figure 5 also shows this, where the first node – the most important split in the data - divides egocentric (0°, 45°, 90°, 270°, 315°) and allocentric (135°, 180°, 225°) views of the hands, Bonferroni corrected *p*<0.001, with a further node within the allocentric views that splits off 180°, Bonferroni corrected *p* = 0.022.

The fixed effects, listed in Table 2, show the estimated change in *d*'*/d*'*_max_* relative to the baseline sensitivity for 0° where *d*'*/d*'*_max_ = *0.85. While performance is marginally better at 45° (+0.05) and at 315° (+0.03), and marginally worse at 90° (−0.04) and at 270° (−0.03), none of these differences reach significance. In contrast, the sharpest falls in sensitivity are seen at the allocentric orientations of 135° (−0.16), 180° (−0.31) and 225° (−0.17), these differences all being significant at p<0.001.

**Table 2:** Effects of orientation on sensitivity.

	Estimate (S.E.)
(Intercept)	0.851 (0.029) ***
angle: 45	0.045 (0.024)
angle: 90	−0.044 (0.032)
angle: 135	−0.164 (0.033) ***
angle: 180	−0.313 (0.047) ***
angle: 225	−0.166 (0.033) ***
angle: 270	−0.027 (0.030)
angle: 315	0.033 (0.025)
Log-likelihood 150	
Deviance −348	
AIC −211	
BIC −54	
N 240	
Groups 30	

***p<0.001.

## Discussion

Consistent with previous studies [2]–[4], [8], [9], we find that response times to judge handedness increase with angle of rotation from 0°, and show particularly sharp increases near 180°. Our stimulus set comprised six hand postures, each shown in eight orientations. We chose highly familiar postures, as familiarity generally gives rise to response time curves that are non-monotonic at 180° [23], allowing us to combine data across postures after excluding the palm posture. We used a mixture of rotations in the picture plane and rotations in depth, recently shown to foster use of motor imagery [20].

Our concern is whether this use of motor imagery persists over the full range of orientations, or whether other strategies (such as employing a preliminary egocentric perspective transformation prior to motor imagery or resorting to a visual object-based transformation) are used for allocentric views of the hands. Others have noted the difficulty in distinguishing mental transformations on the basis of response time data alone [6], an issue we address by using psychophysical sensitivity, *d*', as an additional measure of performance, and by considering both fixed and random effects in our analysis of chronometric data.

While previous studies of the hand laterality task have rarely considered error rates beyond noting that these are typically low and positively correlated with response times, here the pattern of errors - which include ‘misses’ and ‘false alarms’ when the task is conceptualized as a two-alternative forced choice procedure – is used to calculate a measure of sensitivity at each orientation of the hands. We find that sensitivity is consistently high across egocentric views with no significant differences at 0° and at other egocentric views (45°, 90°, 270°, 315°). Sensitivity falls suddenly for more allocentric views (135°, 225°), reaching its lowest point at 180° (Figure 5). Recursive partitioning also shows the primary split in the data to occur between egocentric and allocentric view of the stimuli (Figure 5). While no further splits are seen among egocentric views, a further split occurs among allocentric views, isolating performance at 180°. Interestingly, this primary split in sensitivity between egocentric and allocentric perspectives is mirrored by a corresponding split in the averaged response time data in Figure 3.

Returning to chronometry, we note that experimental effects are usually illustrated using measures of performance that are averaged across participants: this is the case in all previous studies of the hand laterality task with which we are familiar. Mixed effects models allow us to also examine random effects, and here the effects of the participant are paramount. In considering whether two classes of mental transformation have independent neural substrates, Zacks and colleagues [24] noted that, as a consequence of such dissociation, some individuals may excel at one form of mental transformation but be less efficient at another. Specifically, they argue that multiple measures of one type of mental transformation should correlate better amongst themselves than with measures of another type of mental transformation. Extending this logic to the hand laterality task, we suggest that if participants' strategies switch between stimulus perspectives, this switch may reveal itself as a drop in correlation in response times across stimulus perspectives.

The caterpillar plots in Figure 4 are consistent with this. Response times are tightly correlated among egocentric views: as the fixed effects or averaged response times increase with angle of rotation from ‘baseline’ (combined 0°, 45° and 315° in the model), each participant keeps their approximate position in the plot, being somewhat slower than the participant just below them and somewhat faster than the participant just above them. This order begins to break down for allocentric views of the hands at 135° and 225° where correlation with baseline drops to 0.28 and 0.32 respectively. At 180° disorder is more obvious, and correlation drops to 0.01. Considering all six plots, it appears that slower participants, as measured by their performance at baseline, are more likely than faster participants to show a change in strategy.

To summarize, for egocentric views of the hand, our data are consistent with the motor image hypothesis and we provide the usual chronometric evidence: response times increase with increasing rotation of the stimulus hand from 0° with particularly steep increases toward 180°. Our data also provide two novel forms of evidence for the use of motor imagery in the hand laterality task. First, sensitivity is high and unvarying across egocentric views of the hand, indicating a common strategy. Secondly, response times between pairs of egocentric views show high positive correlation, reinforcing the idea that we rotate our hands through an increasingly long ‘mental trajectory’ to reach more extreme stimulus postures.

However, we also find a clear break in sensitivity between egocentric and allocentric views of the hands which is matched by a primary split in the average response time data, suggestive of a change in strategy from that of mental hand rotation. Similarly, response times for allocentric views of the hands (in particular, for 180°) show weaker correlation with response times for egocentric views of the hands, a pattern suggesting that different cognitive processes may be used for different perspectives by a majority, if not by all, participants.

Our data, while suggesting alternative cognitive processes to motor imagery for allocentric views of the hands, are equivocal about what form these may take. In fact, the design of the hand laterality experiment, in which a majority of egocentric postures are combined with fewer allocentric postures, may induce a strategy of motor imagery which simply breaks down or becomes less efficient for those views of the hand for which a first person perspective is less likely to be adopted. Using a different task - but one which also involves making judgments about the spatial configuration of right and left hands - recent research shows that asking people to adopt a first person perspective (imagining their own hand) or a third person perspective (imagining the experimenter's hand) in separate experimental blocks leads mainly to the engagement of motor and visual imagery, respectively [25]. It is possible that in the hand laterality task, too, the presentation of stimuli in egocentric and allocentric views encourages the adoption of first and third person perspectives, respectively. Further studies of the task, in which egocentric and allocentric views are presented in separate experimental blocks, may prove informative. It is also possible that a single, ‘noisy’ mechanism might also account for the data, a solution that does not suppose the operation of different cognitive or neural mechanisms for different views of the hands.

The question of whether motor imagery is used for all orientations in the hand laterality task is not one that we pose in order to undermine the considerable support for the motor hypothesis. Rather it is relevant to the broader issue of how ‘self’ and ‘other’ are distinguished in the brain. Recent research on the extrastriate body area (EBA), an area of lateral occipitotemporal cortex which responds preferentially to images of the human body [26], shows that the right EBA differentiates between views of the body [5], [14]. For stimuli, one of these studies [5] used images of body parts - hands, feet, arms and legs – photographed from both egocentric and allocentric perspectives. Both studies report a significant modulation of EBA activity by stimulus viewpoint such that the right EBA responds more strongly to allocentric than egocentric views. A later study using fMRI adaptation shows that the EBA is also selective to body identity and discriminates images of one's own and others' hands [27]. See also [28]. The EBA appears to be primarily involved in a categorical visual analysis of bodily form [29]; its selectivity to image perspective [5], [14] and to body identity [27], [30] points toward a role in discriminating one's own from other bodies. Egocentric and allocentric views of hand stimuli have subtly different effects as visuomotor primes [31], and motor imagery of hand gestures from a first person or egocentric perspective leads to stronger activation in motor areas than when imagery is from a third person or allocentric perspective [32]. While previous neuroimaging studies of the hand laterality task provide clear evidence for the engagement of the motor system [11]–[13], we suggest that further studies examining contrasts for egocentric and allocentric views of the hands may help elucidate the forms of imagery involved.

Finally, we consider how our results reflect the specific set of postures used in the study. The original set includes images of the palm and of the back of the hand rotated in the picture plane, chosen because almost all previous studies of the hand laterality task use these postures, with some relying exclusively on them [13], [21], [33], [34], [35]. The remaining four postures were chosen for familiarity and include hands reaching to grasp, reaching to shake, pointing with the index finger and making a fist. The stimulus set - which includes hands rotated in depth and in the picture plane, and hands in which the thumb is hidden from view - counters the use of purely visual strategies and satisfies recently suggested criteria for inducing motor imagery [20]. The palm posture has a very different RT-orientation profile than the other five postures, with considerably longer response times for lateral than for medial rotations of the same magnitude. This pattern emphasises the biomechanical constraints of the task and is taken by some to be a hallmark of motor imagery [20]. However, as shown in Figure 2, this profile is not typical of more naturalistic hand movements that are executed in near space. While the palm posture, and other postures which are more difficult to adopt at lateral than at medial orientations, have proved extremely useful in confirming the motoric and kinaesthetic nature of the imagery involved in the hand laterality task, more common and naturalistic postures may prove more useful in answering other research questions. This first pass at using more naturalistic stimuli reveals remarkably similar RT profiles across different postures - pointing, shaking hands and reaching - suggesting that the attribution of hand laterality may be an elementary process that procedes analysis of the social or communicative function of the gestures. However, an examination of the relative magnitude of RT across postures, particularly at egocentric orientations, would be necessary to decide this point.

In conclusion, we find that a measure of psychophysical sensitivity in the hand laterality task, *d*', clearly demarcates task performance for egocentric and allocentric views of the hands. In addition, using mixed effects methods to model the chronometric data we find high positive correlation between response times across egocentric views, but considerably lower correlations between egocentric and allocentric views of the hands. This suggests a common use of motor imagery across egocentric views, but a shift away from motor imagery for allocentric perspectives. Mixed effects models, although still novel in behavioural neuroscience, have previously been applied to chronometric data [36]. Here they provide a means to test for ‘discriminant\validity’, the criterion whereby distinct measures of a specific mental transformation should correlate better amongst themselves than with measures of a different form of mental transformation [24]. We suggest that the shift in performance between egocentric and allocentric views of the hands reflects a natural tendency to adopt a first or third person perspective in the task, which may be ultimately rooted in the early neural encoding of visual images of the body as belonging to oneself or to another person.
